# Comparative analysis of classification techniques for topic-based biomedical literature categorisation

**DOI:** 10.3389/fgene.2023.1238140

**Published:** 2023-11-07

**Authors:** Ihor Stepanov, Arsentii Ivasiuk, Oleksandr Yavorskyi, Alina Frolova

**Affiliations:** ^1^ Knowledgator Engineering Ltd., London, United Kingdom; ^2^ Institute of Molecular Biology and Genetics of NASU, Kyiv, Ukraine; ^3^ Bogomoletz Institute of Physiology, London, Ukraine; ^4^ National Technical University of Ukraine “Igor Sikorsky Kyiv Polytechnic Institute”, Kyiv, Ukraine; ^5^ Department of Mathematics, Kyiv Academic University, Kyiv, Ukraine

**Keywords:** information theory, transformer-based methods, LSTM, unbalanced data, machine learning, text mining, biomedical literature classification, DILI

## Abstract

**Introduction:** Scientific articles serve as vital sources of biomedical information, but with the yearly growth in publication volume, processing such vast amounts of information has become increasingly challenging. This difficulty is particularly pronounced when it requires the expertise of highly qualified professionals. Our research focused on the domain-specific articles classification to determine whether they contain information about drug-induced liver injury (DILI). DILI is a clinically significant condition and one of the reasons for drug registration failures. The rapid and accurate identification of drugs that may cause such conditions can prevent side effects in millions of patients.

**Methods:** Developing a text classification method can help regulators, such as the FDA, much faster at a massive scale identify facts of potential DILI of concrete drugs. In our study, we compared several text classification methodologies, including transformers, LSTMs, information theory, and statistics-based methods. We devised a simple and interpretable text classification method that is as fast as Naïve Bayes while delivering superior performance for topic-oriented text categorisation. Moreover, we revisited techniques and methodologies to handle the imbalance of the data.

**Results:** Transformers achieve the best results in cases if the distribution of classes and semantics of test data matches the training set. But in cases of imbalanced data, simple statistical-information theory-based models can surpass complex transformers, bringing more interpretable results that are so important for the biomedical domain. As our results show, neural networks can achieve better results if they are pre-trained on domain-specific data, and the loss function was designed to reflect the class distribution.

**Discussion:** Overall, transformers are powerful architecture, however, in certain cases, such as topic classification, its usage can be redundant and simple statistical approaches can achieve compatible results while being much faster and explainable. However, we see potential in combining results from both worlds. Development of new neural network architectures, loss functions and training procedures that bring stability to unbalanced data is a promising topic of development.

## 1 Introduction

Drug-induced liver (DILI) injury is an adverse reaction caused by the effect of drugs or another xenobiotic. DILI is a clinically significant condition and one of the reasons for drug registration failures, moreover, it can lead to post-marketing termination and restriction to use. The probability of an individual drug causing liver injury ranges from 1 in 10,000 to 100,000, with some drugs reported as having an incidence of 100 in 100,000 (chlorpromazine, isoniazid) (Devarbhavi, 2012). According to DILIrank - a dataset that consists of 1,036 FDA-approved drugs that are divided into four classes based on their potential for causing drug-induced liver injury (DILI), nearly 18.5% of drugs were classified as most DILI-concern medicines (Chen et al., 2016).

**FIGURE 1:**
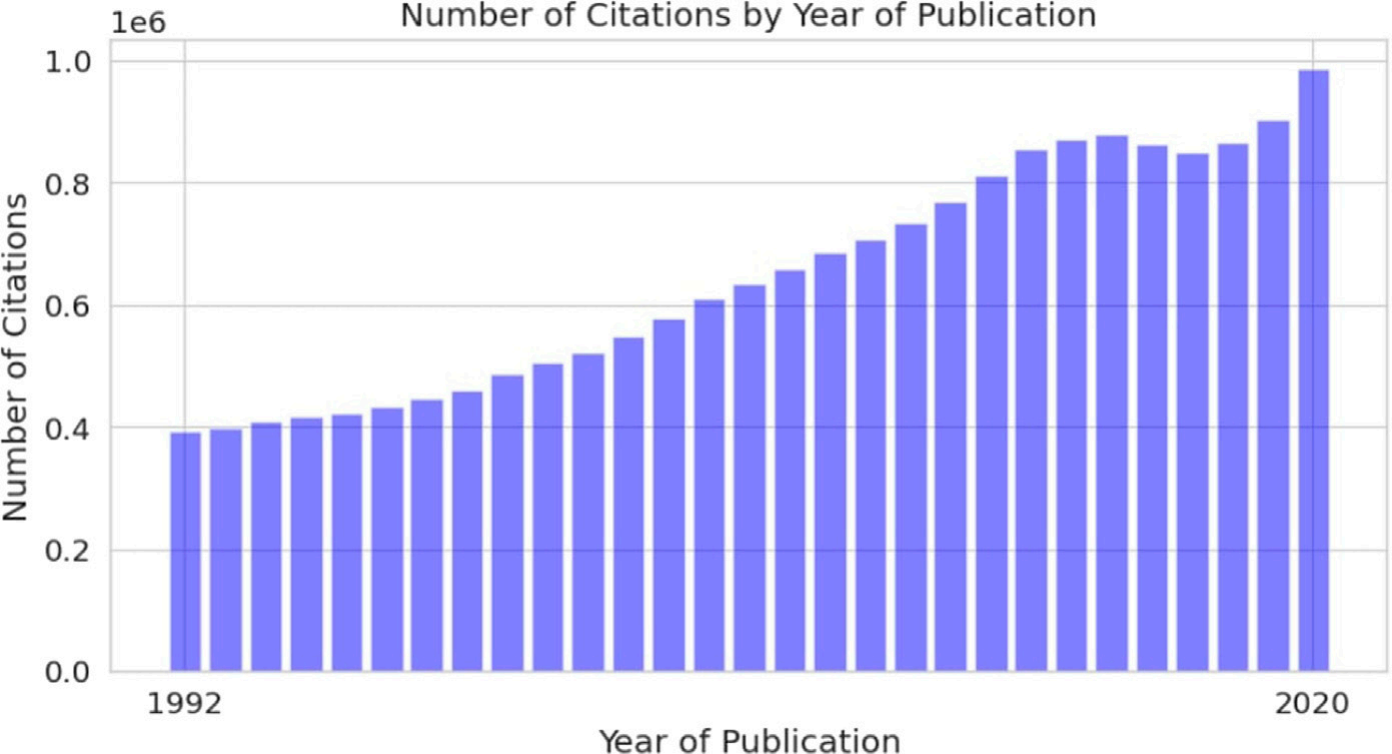
Annual growth of scientific articles in Medline database.

Scientific literature is currently the main source of information related to DILI. However, the number of scientific papers published yearly is growing, making it hard to analyze, not only for individuals but also for large organizations, such as the Food and Drug Administration (FDA). One of the first studies regarding scientific literature production was conducted by De Solla Price, who used publication data collected over the 100 years (1862–1961) to calculate a doubling time. The results showed 13.5 years for doubling the scientific corpus with a 5.1% annual growth rate (de Solla Price, 1965). The development of technologies stimulated accelerated scientific literature production, which made scientific information more accessible but also introduced new challenges. Accessibility of biomedical literature through databases such as Medline and research activity in biomedicine is useful for practically implementing natural language processing (NLP) techniques. In total, Medline has more than 30M citations and more than 800,000 articles added to this database every year (Figure 1) (MED, 2021).

Working with specific biomedical topics, we must consider the sparsity of real-world data related to DILI. To develop, test and compare text classification approaches that can produce high-quality results in sparse corpora, we took part in the “Extended Literature AI for Drug Induced Liver Injury” CAMDA (Critical Assessment of Massive Data Analysis, http://camda.info/) challenge. This challenge provides biomedical publications curated by FDA experts on DILI.

In recent years, there have been significant advancements in NLP, enabling the development of models that can achieve human-level performance on generic tasks such as Q&A on SQUAD 2.0 (Rajpurkar et al., 2016) or student tests. For instance, GPT4 has surpassed the performance of 50% humans in these tasks (OpenAI, 2023). However, most of these models are quite large, resulting in high computational costs when applied to large-scale document processing. At the same time, it is known that real-world use of large and complex models can be quite intricate. Another concern, which relates to the use of complex models is their interpretability. Authors in (Zhan et al., 2021) have used Bag of Words (BOW), Word2Vec (W2V), Doc2Vec (D2V), and TF-IDF approaches to predict ICD codes from related cardiovascular diseases from the outpatient notes with logistic regression. The results showed that TF-IDF dominates other approaches with respect to the AUROC and AURPC values, suggesting that simple and interpretable approaches could and do, in fact, outperform more sophisticated solutions. In a similar research (Zhan et al., 2022) dedicated specifically to DILI, authors leverage the same text processing techniques like BOW, W2V, and TF-IDF, while instead of D2V, Sentence2Vec is used. In addition, the paper includes the use of Random Forests for particular classification in addition to logistic regression. On top of that, authors have implemented ensemble methods to boost the performance of the classification. As it turns out, TF-IDF coupled with logistic regression outperforms other approaches, including the ensembles, with respect to AUROC, Accuracy, AUPRC, and F1 Score metrics. A similar yet unique work on the DILI classification topic is represented by Rathee (2022), where authors are solving the AI-based classifier development problem not only from a theoretical but also from a practical perspective. The paper makes use of Neural Networks-based approaches, as well as classical algorithms, like logistic regression or Support Vector Machines. While the authors used no particular vectorization technique, they mention that for pattern mining, they have used keywords sets, along with their frequencies. As one can presume, this approach produces somewhat similar outputs to TF-IDF.

In our paper, we decided to focus on identifying single words that can determine the class of a document. This means that the requirements for complex contextual understanding are relatively lower compared to tasks like Q&A or text generation. Our research aims to conduct a comparative analysis of various text classification approaches, encompassing transformers, LSTMs (long short-term memory neural networks), information theory, and statistical methods. We will examine their respective advantages and disadvantages, evaluating their performance and speed. Additionally, we have explored methods that enhance the performance of transformers on sparse data, which can be especially valuable for handling contextually complex examples. In summary, our study not only offers practical solutions for tasks like DILI article classification but also provides valuable insights into modern deep learning models and their links to statistical and information theory methods. We’ve explored how various models perform on commonly unbalanced data and introduced techniques for enhancing model performance and handling imbalance. Our research aims to contribute to the development of interpretable, context-aware models suited for real-world, unbalanced datasets.

## 2 Data and methods

### 2.1 Datasets

**FIGURE 2:**
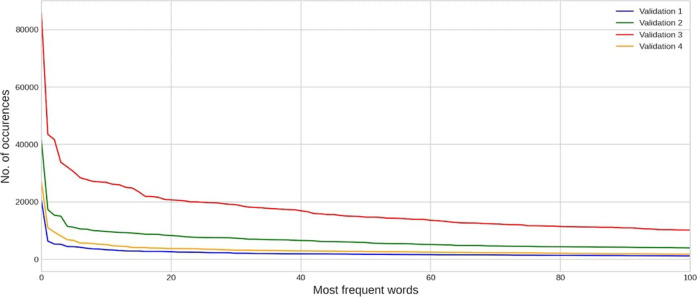
Distribution of the most frequent words in each validation dataset.

CAMDA committee and FDA provided an initial training dataset with approximately 14000 DILI-related papers referenced in LiverTox (Hoofnagle et al., 2013), equally divided into positive and negative examples. Participants of the challenge were also given test and validation datasets that were unbalanced to different degrees, i.e., including more and more true negatives to reflect the difficulty of the real-world task. More precisely, there were 3 test datasets within different subchallenges to which we could submit an unlimited amount of model predictions and, in this way, control the precision of the model (we will describe this effect in the next sections). The first test dataset consists of 4,764 article abstracts, the second one - 21,724 and the third one - 82,753. To test how the model can generalize and be stable to different degrees of negative examples, the validation datasets were added. Participants could make only up to 11 submissions, and in total, we had 4 validation datasets. The first validation dataset consists of 6,494 articles abstracts, the second one - 32,814, and the third one - 100,265. Moreover, to test the over-fitting of models, the fourth Validation dataset consists of 14,000 experts’ summaries of the research instead of extracted articles abstracts. In Figure 2, you can see the distribution of the most frequent words in each validation dataset.

### 2.2 PMI/TF-IDF method

As our first approach to document classification, we used a combination of two statistical methods: pointwise mutual information (Bouma, 2009) and term frequency-inverse document frequency (Havrlant and Kreinovich, 2017), we called this approach PMI-TF-IDF classifier (PTIC). For each example in the dataset, we concatenated Title and Abstract into one block of text, and then we calculated the point-wise mutual information score (PMI) for this block of text:

$$\text{pmi}\left(\text{class},\text{word}\right)=\text{log}\left(\frac{P\left(\text{class},\text{word}\right)}{P\left(\text{class}\right)\cdot P\left(\text{word}\right)}\right)$$ (1)

$$\text{npmi}\left(\text{class},\text{word}\right)=-\frac{\text{pmi}\left(\text{class},\text{word}\right)}{\text{log}\left(P\left(\text{class},\text{word}\right)\right)}$$ (2)

Where *P*(class) - is the class probability in the dataset (fraction of examples of this class in the dataset), *P*(word) - word frequency in the dataset, *P*(class, word) - word frequency in the examples of the given class. npmi is the normalized pmi, we interpret it as the importance of a word for the class. Then we calculate term-frequency/inverse-document frequency:

$$\text{tf}=1/n,\;\text{idf}=\text{log}\left(\frac{N}{N_{\mathrm{word}}}\right),\;\text{tf}-\text{idf}=n_{\mathrm{word}}\cdot \text{tf}\cdot \text{idf}$$ (3)

Where *n* - is the number of words in the given text, *N* - is the number of documents (i.e., examples). *N*_*word*_ - is the number of documents that contain this word, *n*_*word*_ - the number of times a given word is present in the given document.

We decided to calculate the final decision score by multiplying PMI and TF-IDF values for each word and class. TF-IDF measures the importance of a word in a document, giving higher values for rare words in a document corpus but frequent in concrete text passages. PMI assesses a word’s importance to a specific class, peaking when the word is uncommon but frequent within the class.

To make a decision about the class, we calculated the sum of pmi ⋅tf-idf for all words in the text. First, we calculated a score for the PMI of a word with the first class and then with the second class. The decision about the class of a document was based on the highest sum.

$$F\left(C\right)=\overset{n}{\underset{i=1}{\sum }}{\text{tf}-\text{idf}}_{\mathrm{word}}\cdot \text{npmi}\left(C,{\text{word}}_{i}\right)$$ (4)

$$C_{\mathrm{pred}}=\text{argmax}\;F\left(C\right)$$ (5)

For the division of the text into words, we used tokenization tools from the NLTK Python library: we filtered stop words, chose words that consisted of alphabetical characters only, and converted everything to lowercase.

Because PMI is not directly designed for the classification tasks, for the current CAMDA challenge, we decided to add additional weights trained using gradient descent. It can be viewed as a logistic regression type of model, and we call it logit PTIC. In Eq. 6, you can see a variant for binary classification, but it can be easily adapted for multiclass classification.

$$F=Sigmoid\left(\overset{n}{\underset{i=1}{\sum }}\overset{c}{\underset{j=0}{\sum }}tf-idf\left(C,i\right)\cdot npmi\left(C,i\right)\cdot Wci\right)$$ (6)

$$C_{\mathrm{pred}}=argmax\;F\left(C\right)$$ (7)

where *C* - is one of the classes; *C*_*pred*_ - predicted class; *n* - number of words; *c* - number of classes; *Wci* - trained weight using gradient descent.

### 2.3 Naïve Bayes classifier

Naïve Bayes classifier is based on the Bayes theorem that describes how we can estimate the posterior probability of event A given event B based on some conditions.

$$P\left(A|B\right)=\frac{P\left(B|A\right)P\left(A\right)}{P\left(B\right)}$$ (8)

In our case, event A can be interpreted as text class and event B as some words in the document. It is called Naïve because we make assumptions that the probability of getting some word in the text or feature, in general, is independent of another word. In this case, the equation for calculating the probability of some classes looks the following.

$$P\left(c|x_{1},\ldots ,x_{n}\right)\propto P\left(y\right)\overset{n}{\underset{i=1}{\prod }}P\left(x_{i}|c\right)$$ (9)

To select the predicted class, we simply select the class with the higher probability after logarithmization, the final formula looks like this:

$$C=\arg\underset{c}{\max}\left(\log\left(P\left(c\right)\right)+\overset{n}{\underset{i=1}{\sum }}\log\left(P\left(x_{i}|c\right)\right)\right)$$ (10)

In this section, we would like to compare PTIC with Naïve Bayes. While PMI and Naïve Bayes look similar at first glance, they have certain distinctive features. Because in extreme cases, due to logarithmization, PMI and Naive Bayes can approach −*∞* when *p*(*x*, *y*) = 0. This issue affects scaling, result reproducibility, model optimization, and real-world usage. The reasons listed before suggest the need for some other forms of PMI, one such PMI sub-type is the Positive PMI, as defined below.

$$ppmi\left(x,y\right)=\max\left(\log\frac{P\left(x,y\right)}{P\left(x\right)P\left(y\right)},0\right)$$ (11)

In this work, we saw higher utility in using Normalized PMI compared to Positive. The main advantage of the Normalized version lies in its boundness to the [−1, 1] range. These bounds provide a natural explanation of values that can be obtained, where −1 (or 1) implies that events never occur together (or occur consistently), and 0 indicates independence. Clearly, an additional benefit of normalization is that we can soften the ‘outliers’, and the obtained values are more well-behaved. Compared to Bayes, such bounds provide a more straightforward interpretability of results.

One can easily represent PMI and its Normalized version in Bayesian terms as follows.

$$pmi\left(A;B\right)=\log\frac{P\left(A|B\right)}{P\left(A\right)},$$ (12)

$$pmi\left(B;A\right)=\log\frac{P\left(B|A\right)}{P\left(B\right)}$$ (13)

Next, to show the difference between the PMI-based and Bayesian approaches for classification, we can represent both equations in terms of the Shannon information *h*(*x*) = − *log* (*p*(*x*)).

$$pmi\left(A;B\right)=h\left(A\right)+h\left(B\right)-h\left(A,B\right)$$ (14)

$$npmi\left(A;B\right)=\frac{h\left(A\right)+h\left(B\right)-h\left(A,B\right)}{h\left(A,B\right)}$$ (15)

Now, we can substitute this into Eqs 4, 10 to get the following representations with the omission of indexes for better readability.

$$C_{\mathrm{npmi}}=argmax\left(\sum tf-idf\cdot \frac{h\left(A\right)+h\left(B\right)-h\left(A,B\right)}{h\left(A,B\right)}\right)$$ (16)

$$C_{\mathrm{Bayes}}=argmax\left(-h\left(A\right)-\sum h\left(A\right)+h\left(B,A\right)\right)$$ (17)

Here, for Bayes equation we represent *log* (*P* (*x*_*i*_|*c*)) as $\log\left(P\left(x_{i},c\right)\right./P\left(c\right)$ . As one can observe, from the Shannon information’s point of view, these two approaches do not have much in common. Indeed, the final class value in the suggested method is computed more carefully than with classical Naïve Bayes. It is worth mentioning that the PTIC classifier is affected by *tf* − *idf*, which implies that, for certain cases, the behaviour of this approach can be limited. Since the *npmi* term is restricted in the magnitude of the values, the *tf* − *idf* term can have a greater effect than it is desirable under certain circumstances.

### 2.4 Transformers

Transformers are a type of neural network architecture that gained significant popularity in natural language processing (NLP) tasks. The Transformer model was introduced in a 2017 paper titled “Attention is All You Need” (Vaswani et al., 2017). Since then, it has become the *de facto* standard for many NLP tasks, including machine translation, text summarisation, question answering, and language generation. The core idea behind Transformers is the self-attention mechanism, which allows the model to weigh the importance of different words in a sentence when processing each word. This is implemented by generating Keys, Queries, and Values matrices for each document.

$$\text{Attention}\left(Q,K,V\right)=\text{softmax}\left(\frac{QK^{T}}{\sqrt{d_{k}}}\right)V$$ (18)

where:

• **Q** represents the query matrix.
• **K** represents the key matrix.
• **V** represents the value matrix.
• *d*_*k*_ is the dimension of the key matrix.

The attention mechanism computes the compatibility between the query matrix **Q** and the key matrix **K** by taking their dot product and scaling it by the square root of the key dimension *d*_*k*_. The result is then passed through a softmax function to obtain attention weights. Finally, these attention weights are multiplied element-wise with the value matrix **V** to obtain the final attention output. All of this is done in parallel by generating different matrices that convert the input matrix to keys, queries, and values. This mechanism enables Transformers to capture long-range dependencies and relationships between words more effectively than traditional recurrent neural networks (RNNs) or convolutional neural networks (CNNs).

In our work, we used 3 different architectures of transformers. Encoder-based transformers use a bidirectional attention mechanism, which means some token receives information from left and right tokens. Such architecture is used mainly for comprehension tasks, such as text classification, NER, Q&A, *etc.* We decided to test two types of pre-trained encoder models: SciBERT (Beltagy et al., 2019) and Longformer (Beltagy et al., 2020). The main difference is the number of tokens that can be used as input to the model. 512 tokens - is the standard limit for BERT models (Devlin et al., 2019), but our dataset has many examples of more than 512 tokens. Also, an essential difference between these transformers is that Longformer utilizes sliding window attention when only a limited number of token embeddings are selected for attention.

On the contrary, decoder-based models use uni-directional attention and are auto-regressive for various text generation tasks, predicting the next tokens. We used BioGPT as representative of this class of models, which was trained on biomedical articles and demonstrated state-of-the-art performance on various biomedical Q&A (Luo et al., 2022).

Encoder-decoder models were the first architecture proposed as transformers and are used for text-to-text tasks such as translation. These models contain separate encoder and decoder blocks. We used the Flan-T5 (Chung et al., 2022) - fine-tuned version of the T5 (Raffel et al., 2019) model on more than one thousand different tasks as representative of such a class of models. In comparison to (Vaswani et al., 2017) it has Layer Norm bias removed, placing the layer normalization outside the residual path; moreover, it used a different positional embedding schema - instead of sinusoidal position signal or learned position embeddings, relative position embeddings (Shaw et al., 2018) was applied. T5 was trained on the Colossal Clean Crawled Corpus, which is a large (760 GB) cleaned corpus of English texts.

The characteristics of all used transformers for comparison on given tasks are described in Table 1.

**TABLE 1:** Characteristics of used transformers.

Characteristic	SciBERT	Longformer	BioGPT	T5-base
Architecture	Encoder	Encoder	Decoder	Encoder-decoder
Position encoding	Trainable	Trainable	Trainable	Relative
Attention mechanism	Bidirectional dense	Sliding-window	Unidirectional-dense	Dense with relative encoding
Amount of parameters	109M	148M	346M	770M
Pretraining corpus	Scientific	Generic	Biomedical	Generic

### 2.5 LSTM

Before transformers, LSTM (Hochreiter and Schmidhuber, 1997) was the standard for language modelling. Initially, it was designed to learn long-term dependencies in time-series data and to solve the vanishing gradient problem that is so frequent in classical RNNs. LSTM uses memory cells, which allow the network to store and access information over extended periods of time. Each memory cell has three main components: an input gate, a forget gate, and an output gate. The input gate determines how much of the new input information should be stored in the memory cell. The forget gate controls what information should be discarded from the memory cell. The output gate determines how much of the memory cell’s content should be output at each time step. By utilizing these gates, LSTM networks can selectively store and access past information, which helps preserve long-term dependencies.

The first time LSTM was used for language modelling was in the work of Sundermeyer et al. (Sundermeyer et al., 2012), where it showed much better performance than classical RNNs. If we compare LSTM with transformers, one of the main advantages of the last is the ability to parallelize computations that can significantly increase training and inference speed. To get the hidden representation of the next token in the sequence, LSTM requires the representation of the previous token, on the contrary, in transformers, the representations of tokens are computed in a parallel manner. However, the sequential nature of LSTM leads to the limited ability of further tokens to influence the current token due to the distillation of information moved from token to token, which can lead to a drop in accuracy, especially for context-dependent tasks with long-distance dependencies. But if we talk about the advantages of LSTM compared to transformers, we can infer that LSTM is not limited to the sequence size, which is an issue related to architectures that use learned positional embeddings with fixed sizes. However, such an issue was solved using relative positional encoding with limited effect on performance.

## 3 Experimental setups

To realize our experiments, firstly, we divided the initial training dataset into training and testing subsets, the last one accounting for 10% of the initial data. Our experiments were conducted on an Ubuntu 22.04.2 LTS machine with an Intel(R) Xeon(R) CPU @ 2.20 GHz. We used Nvidia T4 GPU to train deep learning models.

While experimenting with PTIC, we choose 5 as a minimal amount of times a word occurs in the training subset. Additionally, we filtered the corpus from stop words to limit the effect of noise from this category of words. In the case of the Naive Bayes Classifier, we selected the same strategy and minimal count of a word in a class. What about the logistic regression model of PTIC we trained it for 1,000 epochs with a batch size equal to 100. As the optimization algorithm was selected, Adam (Kingma and Ba, 2014), we chose the learning rate to be equal to 1 × 10^−5^

In the case of transformers, we used the HuggingFace transformers (Wolf et al., 2019) to access the model’s weight as well as their APIs for training models. As a backend, PyTorch (Paszke et al., 2019) was chosen. For all tested models except Longformer, we selected the maximum length of tokens input equal to 512 tokens, while for Longformer, the value was chosen to be equal to 1,024 tokens. In all cases, we used the AdamW algorithm for optimization (Loshchilov and Hutter, 2017). The learning rate for encoder-based models was equel to 2 × 10^−5^, for BioGPT and T5, it was 1 × 10^−5^. Weight decay was equal to 0.001, and the epsilon - term added to the denominator was equal 1 × 10^−8^. For SciBert, the batch size was equal to 12, while for Longformer, due to the larger context, the size was equal to 6. In the case of T5, we used flan-T5-base, batch size was equal to 5 and learning rate was 1 × 10^−4^, Seq2SeqTrainer from HuggingFace transformers were used as training pipeline. What about the decoder-based model we used BioGPT large version, which was trained using HuggingFace Trainer for 3 epochs with batch size 2 and learning rate 1 × 10^−4^. In terms of LSTM, we used a pre-trained tokenizer of the SciBert uncased version. The token embedding size was chosen to be equal to 768, while the hidden embedding size was equal to 256. We trained to the version of LSTM one of them was initialized with SciBert token embeddings that were trained for 3 epochs with a learning rate equal to 1 × 10^−4^ and batch size equal to 16. The uniformly initialized version of a model was trained for 10 epochs with 1 × 10^−3^ and the same batch size as the previously described version.

## 4 Results

In this section, we will describe the performance of our PMI/TF-IDF, Naïve Bayes classifiers and transformers such as SciBERT, Longformer, BioGPT and T5 on Validation datasets. In Table 2, you can find accuracy, F1 score, precision and recall for Validation Set 1. We can see that Longformer demonstrates the best performance due to its ability to process long sequences. The logistic regression version of PMI/TF-IDF performs on 2% of the F1 score better than the standard one.

**TABLE 2:** Performance of the tested models on Validation Set 1.

Metrics	PTIC	logit PTIC	Naïve Bayes	Longformer	FlanT5-base	BioGPT	LSTM	SciBert
Accuracy	0.8677	0.9039	0.8501	0.9454	0.9146	0.9029	0.845	0.9031
F1-score	0.8804	0.9039	0.8684	0.9485	0.9223	0.9097	0.8454	0.9018
Recall	0.7928	0.9236	0.9511	0.965	0.9736	0.9401	0.8151	0.8560
Precision	0.8302	0.8949	0.7989	0.9324	0.876	0.8811	0.8781	0.9528

On Validation Set 2 (Table 3), we observe an opposite situation, where Longformer significantly dropped in performance, even though our statistical information-based approach demonstrates better performance. We will analyze this effect in the next section. On this dataset, SciBERT, the model that was trained on domain-specific data, demonstrates the best performance.

**TABLE 3:** Performance of the tested models on Validation Set 2.

Metrics	PTIC	logit PTIC	Naïve Bayes	Longformer	FlanT5-base	BioGPT	LSTM	SciBert
Accuracy	0.956	0.9604	0.9031	0.9458	0.8782	0.9516	0.9151	0.9678
F1-score	0.7877	0.8174	0.6688	0.7477	0.6219	0.7997	0.661	0.8455
Recall	0.9372	0.8621	0.9517	0.7809	0.9739	0.9398	0.8046	0.8565
Precision	0.7828	0.7771	0.5156	0.7171	0.4568	0.6959	0.5609	0.8347

In comparison to Validation Set 2, the third dataset contains even more negative examples, being more unbalanced. As we can see from the results presented in Table 4, the trend is the same as in the case of Validation Set 2. FlanT5-base has the worst performance, being completely unstable on an unbalanced dataset. Naïve Bayes also demonstrated a significant drop in performance, which can be explained by the different probabilities of negative classes in training and validation. Surprisingly, our simple approach outperformed complicated models such as transformers. However, in the next chapter, we will discuss possible tips on how to adapt neural networks to unbalanced situations.

**TABLE 4:** Performance of the tested models on Validation Set 3.

Metrics	PTIC	logit PTIC	Naïve Bayes	Longformer	FlanT5-base	BioGPT	LSTM	SciBert
Accuracy	0.9763	0.9809	0.9262	0.9717	0.8824	0.9613	0.9552	0.9448
F1-score	0.6932	0.7352	0.4649	0.6267	0.3582	0.6208	0.5153	0.511
Recall	0.7924	0.7853	0.9514	0.7036	0.9736	0.9402	0.5067	0.8564
Precision	0.616	0.6912	0.3076	0.565	0.2194	0.4634	0.7065	0.3642

We also observe an interesting situation with Validation Set 4 (Table 5), which is nearly balanced but has another type of document than presented in the training dataset. These results can demonstrate the ability of the model to generalize. Our introduced model demonstrated the best performance on this set of models, achieving a 0.9398 F1 score, which is even bigger than on the Validation Set 1. It can be explained because the last set depends more on concrete words that determine the “DILI” class.

**TABLE 5:** Performance of the tested models on Validation Set 4.

Metrics	PTIC	logit PTIC	Naïve Bayes	Longformer	FlanT5-base	BioGPT	LSTM	SciBert
Accuracy	0.871	0.9397	0.8451	0.9097	0.9022	0.9093	0.9552	0.9232
F1-score	0.8835	0.9398	0.8641	0.9091	0.9096	0.9164	0.5153	0.925
Recall	0.9783	0.9407	0.9847	0.9026	0.9836	0.9941	0.5067	0.9459
Precision	0.8055	0.9388	0.7698	0.9157	0.8459	0.85	0.7065	0.905

Additionally, we have tested our developed method - PTIC, on a more context-dependent binary classification dataset IMDB50k (Maas et al., 2011) with different dictionary sizes. Overall, the performance on IMDB50k is less than on the DILI dataset, however, the nature of dependency between dictionary size and performance is relatively the same, as can be seen in Supplementary Figure S1. Also, we have tested a related parameter, but from another angle, in Supplementary Figure S2, you can see dependencies between a minimal count of words to be included and accuracy, precision, recall and F1 score.

Because the training datasets can be biased, we decided to check the stability of PTIC during the different fractions of incorrect examples in the training set. Results show that the method is pretty robust and begins to lose its accuracy only after 28% of incorrect examples are introduced (Supplementary Figure S3).

## 5 Discussion

To understand the drop in performance of the models in terms of F1 score, we need to understand how the score is calculated. The F1 score is the harmonic mean of precision and recall.

$$F1=\frac{2\cdot \text{precision}\cdot \text{recall}}{\text{precision}+\text{recall}}$$ (19)

Recall is the rate of retrieved true positives to all true positives and false negatives. Precision is the rate of true positives to all entries predicted as positive. In our case, when we increase the number of negative examples and the probability of false negatives is the same, we just get absolutely more false negative examples, which leads to a drop in precision. One way to decrease the chance of getting false positives is to move the threshold of considering prediction as positive. It will lead to an increase in false negatives, however, in the case of an unbalanced dataset with more negative examples, we will get a higher F1 score. But, as one can assume, different models have different distances on the scores scale for true positives and false positives, basically, it will influence how close we can achieve performance similar to balanced datasets. The receiver operating characteristic curve is one way to estimate the relation between the rate of false positives and true positives.

From the results in the previous section, we can see that neural networks are unstable on unbalanced datasets, and they tend to consider that the probability of positive and negative classes is the same. We decided to try focal loss, first used in computer vision for object detection in sparse space. Focal loss focuses training on a sparse set of hard examples and prevents many easy negatives from overwhelming the classification during training.

$$FL\left(p_{t}\right)=-\alpha_{t}{\left(1-p_{t}\right)}^{\gamma }\log\left(p_{t}\right)$$ (20)

• *p*_*t*_: the probability of the true class
• *α*_*t*_: balancing factor for class *t*
• *γ*: focusing parameter

In our experiments, the *γ* factor was equal to 2 when *α*_*t*_ for the positive class was 0.3.

**FIGURE 3:**
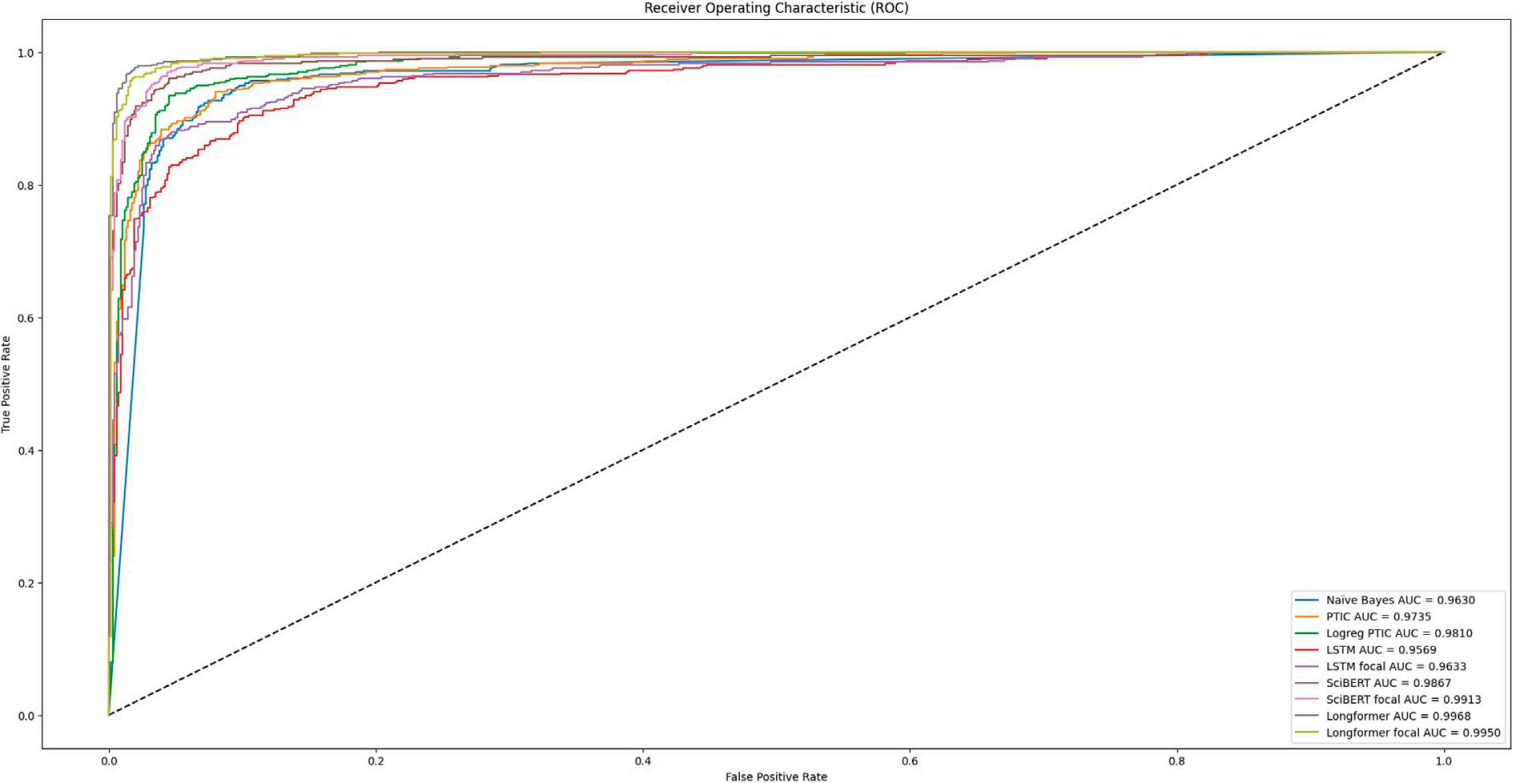
Reciver operating characteristics of tested models during testing on initial dataset.

Figure 3 can explain many of the models’ performances. It visualizes the dependency between the true positive rate and the false positive rate at different models’ decision thresholds. This curve was obtained after analysis of model predictions on the initial dataset, which was split into train and test sets. We can see that training with focal loss can increase AUC (area under the ROC curve) for almost all tested models except Longformer. For such models, we need fewer false positive results to achieve a better true positive rate, which means that we will have higher precision on unbalanced datasets. Our experiments on Validation datasets show that the performance of models trained with focal loss significantly expanded for SciBert, especially for Validation set 3, by nearly 14.

It is important to characterize another type of architecture of transformers we used - T5, more precisely flan-T5-base, and BioGPT large. Because the task for this architecture was set as text generation, it is overcomplicated for such cases as topic classification and makes it harder to tune on unbalanced datasets. However, recently, such models have been actively used in an instructional manner and can be used as zero-shot classification, which makes it a good choice for situations where we have limited training data.

Because we trained LSTM from scratch, it misses generic language information, which transformers get during pre-training. We decided to train LSTM with the tokenization schema of SciBert, taking its pre-trained token embeddings that already encode generic and domain-specific knowledge. The performance is increasing for each validation dataset, as shown in Supplementary Figure S4, and more surprisingly, for Validation set 4, we got the best results not only in the frame of our models but in the challenge itself. The figure demonstrates an increase in the F1 score for each validation dataset.

Finally, we would like to discuss our developed method PTIC. The fundamental nature of this method makes it basically limited to topic classification tasks, however, when applied to such problems, the model demonstrates competitive performance with modern transformers. Based on the results demonstrated in Supplementary Figure S1, we can say that for sentiment analysis tasks that are more context-dependent, we need a much larger dictionary size to obtain competitive results. In terms of our topic classification task, we can approve that a limited amount of words determines a positive class. It was visually shown in Supplementary Figure S2 that with an increase in minimal word count, we get a small increase in Recall and a significant drop in Precision. It tells us that words determined by positive class are frequent in the dataset and more influential on the model decision, and during a decrease in dictionary size, we lose important words that can be related to negative class.

## 6 Conclusion

The field of topic classification often does not require complex models. In this study, we conducted a comparative analysis of various classification techniques, starting from Naïve Bayes and neural networks and concluding with introducing the PMI-TF-IDF classifier (PTIC). Our results demonstrate that PTIC exhibits robust performance, high speed, and balanced precision and recall, making it particularly suitable for handling unbalanced scenarios. This method can serve as an initial approach for text classification, allowing for identifying influential words and assessing context dependency in classification problems. Furthermore, we enhanced our method by incorporating additional training weights for individual words, which, while reducing interpretability, significantly improved accuracy. Our findings indicate that the ability to process long sequences of arbitrary length is crucial when classifying text based on specific information scattered throughout the text. It means that transformer models, such as Longformer, get advantages in such cases due to their ability to handle long sequences. Additionally, pretraining on domain-specific or generic corpora enhances performance, and utilising focal loss effectively addresses imbalanced datasets. During our research, we were not able to create a general model that can handle each dataset, however, our results can be helpful in future attempts to create long-context aware models that can be stable to extreme unbalance of data. The code of PTIC is available in the following repository—https://github.com/sysbio-vo/ptic.

## Data Availability

Publicly available datasets were analyzed in this study. This data can be found here: http://camda2022.bioinf.jku.at/contest_dataset#extended_literature_ai_for_drug_induced_liver_injury. All additional datasets used for testing, as well as plots, notebooks, and submissions of predictions, can be seen via the link: https://github.com/sysbio-vo/article-ptic-suppl.
