# Supplementary material for: Comparative analysis of classification techniques for topic-based biomedical literature categorisation
Source: Front Genet. 2023 Nov 7;14:1238140. doi: 10.3389/fgene.2023.1238140 (PMC10668010; doi:10.3389/fgene.2023.1238140)
Supplement: Supplementary file 1 [file DataSheet1.PDF]

Supplementary Material

1 SUPPLEMENTARY FIGURES

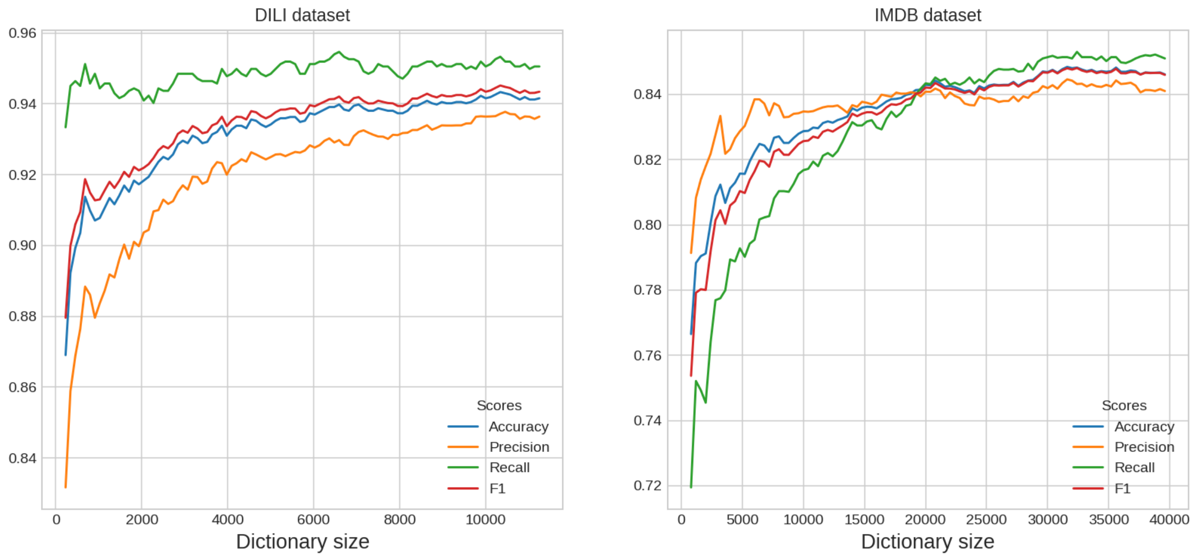

Figure S1. Dependencies between performance and dictionary size on DILI initial and IMDB datasets

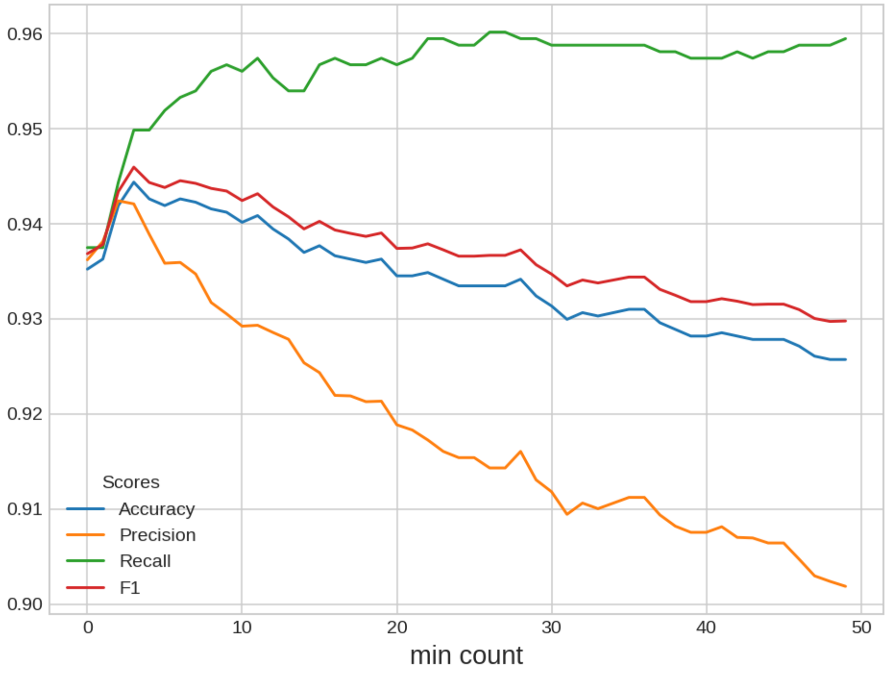

Figure S2. Changes in performance depending on word minimal count parameter

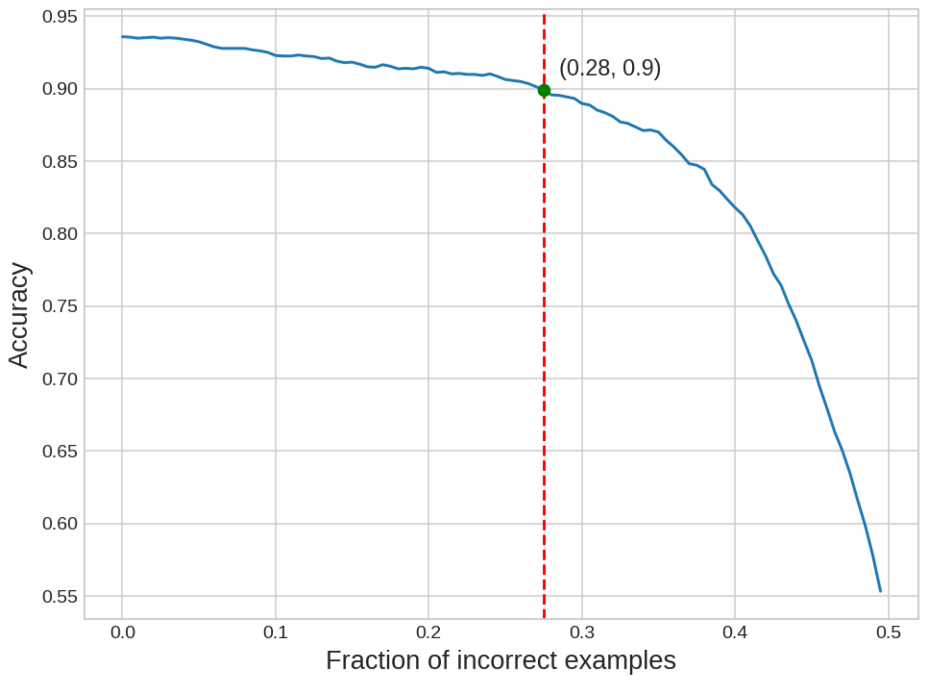

Figure S3. Changes in performance depending on portion on incorrect labeled training data

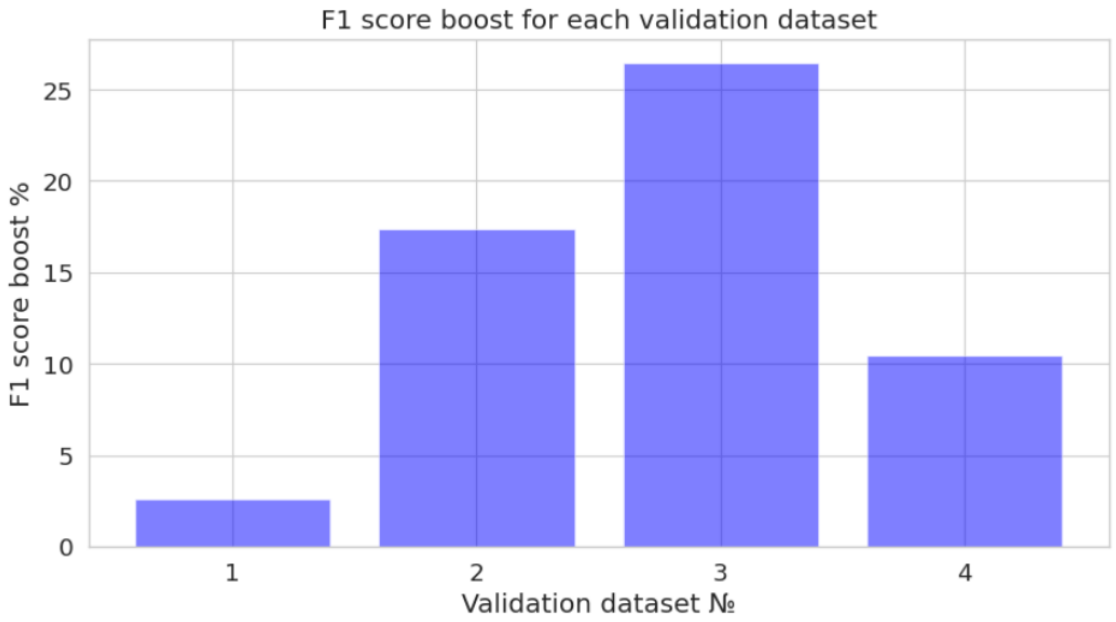

Figure S4. F1 score difference between LSTM with SciBERT word embeddings and randomly initialized for each validation dataset.
